# Correlation of WWOX, RUNX2 and VEGFA protein expression in human osteosarcoma

**DOI:** 10.1186/1755-8794-6-56

**Published:** 2013-12-15

**Authors:** Jilong Yang, Linru Zhao, Wei Tian, Zhichao Liao, Hong Zheng, Guowen Wang, Kexin Chen

**Affiliations:** 1 Department of Bone and Soft Tissue Tumor, Tianjin Medical University Cancer Hospital & Institute, Tianjin 300060, China; 2 Epidemiology and Biostatistics, Tianjin Medical University Cancer Hospital & Institute, Tianjin 300060, China

**Keywords:** Osteosarcoma, WWOX, RUNX2, VEGFA, aCGH,FISH, Gene amplification

## Abstract

**Background:**

To investigate associations between WW domain-containing oxidoreductase (WWOX), runt-related transcription factor 2 (RUNX2) and vascular endothelial growth factor alpha (VEGFA) in human osteosarcoma (OS).

**Methods:**

Copy number aberrations of *WWOX*, *RUNX2*and *VEGFA* genes were detected by microarray comparative genomic hybridization (aCGH) in 10 fresh OS tissue samples. *VEGFA* gene alterations were also investigated and validated by fluorescence in situ hybridization (FISH) in 54 formalin-fixed and paraffin-embedded (FFPE) OS samples. Protein expression of WWOX, RUNX2 and VEGFA were examined in 54 FFPE OS samples by immunohistochemistry (IHC).

**Results:**

Analysis of previously published OS aCGH data (GSE9654) and aCGH data from this study (GSE19180) identified significant deletion of *WWOX* in 30% (6/20) of OS samples, whilst significant increase in both *RUNX2* and *VEGFA* gene copy numbers were detected in 55% (11/20) and 60% (12/20) of OS samples, respectively. FISH demonstrated increased *VEGFA* gene copy number in 65.9% (31/47) of evaluable samples, in either focal or large fragment forms. Compared with positive expression of WWOX in 38.9% of the OS samples, positive expression of RUNX2 and VEGFA protein was found in 48.1 and 75.9% of samples. Although there was no significant association between gene copy number aberration and protein expression for WWOX and RUNX2, significant positive correlation between increased *VEGFA* gene copy number and VEGFA protein expression was observed. Although there was no significant reverse association between WWOX and RUNX2 expression, a significantly positive relationship was observed between RUNX2 and VEGFA protein expression.

**Conclusions:**

Our data show increased *RUNX2* and *VEGFA* gene copy numbers and elevation of their respective proteins in human OS. Positive correlation of RUNX2 and VEGFA suggests that both increased *VEGFA* gene copy number and RUNX2 overexpression facilitate increased expression of VEGFA.

## Background

Osteosarcoma (OS) is the most common, primary, malignant bone tumor within the non-hematopoietic system. OS frequently occurs in the metaphysis of actively growing long bones and is characteristic of short and rapid progression. It has high incidence of pulmonary metastasis and poor prognosis, and mainly affects children and adolescents [1]. Because of a complexity of karyotypes and a highly unstable genome, OS usually exhibits both numerical and structural chromosomal alterations [2]. As a putative tumor suppressor gene, *WWOX* is located at chromosome 16q23.3-q24.1, spanning common fragile site FRA16D [3]. *WWOX* is detected as functional loss or frequent attenuation of protein expression in combination with poor prognosis. This often results from abnormal mRNA splicing of WWOX, missing exons, loss of heterozygosity (LOH) and hypermethylation in numerous carcinomas [4-9].WWOX might play tumor-suppressor function through interaction with TNF, p53, Bcl-2, ErbB-4 and c-Jun [3,10-12]_._ A recent study indicated that WWOX was physically and functionally associated with RUNX2 and can suppress RUNX2 transactivation by interaction between the first WW domain and RUNX2 [13]. In addition, absence of WWOX seems to contribute to increased RUNX2 expression, further affecting bone growth and metabolism, initiating OS tumorigenesis [14].

The RUNX2 region (6p12-21) is often detected in OS using gene amplification and protein overexpression, suggesting upregulation of this gene and its protein is associated with tumorigenesis, progression, metastasis and unfavorable outcome [15-21]. Importantly, Kyle and colleagues observed an inverse relationship between WWOX and RUNX2 expression in WWOX-deficient mice and OS cell lines [14]. Additionally, RUNX2 is a critical element for VEGF mRNA transcription and protein expression in tumorigenesis [22]. Aqeilan and colleagues found that ectopic expression of WWOX in MDAMB231 breast cancer reduced expression of RUNX2 and its target genes, including VEGF [13]. However, little is known about the correlation of WWOX, RUNX2 and VEGF in human OS tissues. In this study, we observed *WWOX*, *RUNX2* and *VEGFA* gene copy number status and protein expression levels using microarray comparative genomic hybridization (aCGH), immunohistochemistry (IHC) staining and fluorescence in situ hybridization (FISH), in order to investigate correlations between these components.

## Methods

### Clinical information for OS tissues

Ten fresh OS tissue biopsies were obtained for aCGH analysis. Fifty-four formalin-fixed and paraffin-embedded (FFPE) OS tissues were obtained for FISH and IHC analysis (including nine aCGH analysis samples. One case which had aCGH data was excluded from further assay because of no enough sample). Clinicopathological characteristics comprised age, sex, pTNM stage, recurrence and metastasis (Table 1). Disease-free and overall survival rate ranged from 0 to 94 months, with medians of 7.5 and 12 months, respectively. All samples and clinical data were obtained and analyzed at Tianjin Medical University Cancer Institute and Hospital, China. The study and all procedures were approved by the Institutional Review Board (IRB) at Tianjin Medical University Cancer Institute & Hospital (TMUCIH).

**Table 1:** RUNX2 and VEGFA protein expression level and their correlation with clinical pathologic factors

**Characteristics**	**RUNX2**					**VEGFA**				
	**-**	**+**	**++**	**+++**	***P *** **value**	**-**	**+**	**++**	**+++**	***P *** **value**
Sex										
Male (26)	15	4	1	6	0.435	10	5	4	7	0.012*
Female (28)	13	9	2	4		3	17	2	6	
Age groups										
≤15 y (14)	8	3	0	3	0.027*	4	6	0	4	0.730
15–20 y (22)	11	7	2	2		5	9	2	6	
21–30 y (9)	5	2	0	2		1	4	2	2	
31–40 y (1)	0	0	1	0		1	0	0	0	
>40 y (8)	4	1	0	3		2	3	2	1	
PTNM stage										
I stage (4)	2	1	0	1	0.906	0	3	1	0	0.168
II stage (18)	8	4	3	3		5	5	1	7	
III stage (2)	2	0	0	0		0	1	1	0	
IV stage (1)	1	0	0	0		1	0	0	0	
Recurrence										
No (47)	26	11	2	8	0.484	13	20	4	10	0.132
Yes (7)	2	2	1	2		0	2	2	3	
Metastasis										
No (35)	19	8	2	6	0.951	11	14	3	7	0.351
Yes (11)	5	3	1	2		1	4	2	4	
Disease free survival (mean months ± sd)	Log Rankχ^2^ = 1.331 (60.238 ± 7.881)				0.722	Log Rankχ^2^ = 6.277 (60.238 ± 7.881)				0.099
Overall survival (mean months ± sd)	Log Rankχ^2^ = 0.984				0.805	Log Rankχ^2^ = 0.471 (70.903 ± 6.737)				0.925

*Significantly different *P* < 0.05.

### aCGH investigation and data analysis

aCGH was performed as previously described [23]. Labeled genomic DNAs were hybridized using the Agilent Human Genome CGH Microarray (4 × 44 k) (Agilent Technologies, Palo Alto, CA).Genomic DNA was isolated according to standard procedure. These arrays represent over 43,000 coding and non-coding human sequences, yielding an average 35kbp oligonucleotide probe spatial resolution. At least one target sequence was measured for every characterized gene, and known cancer genes were measured using a minimum of two probes. Probes were designed based on the University of California Santa Cruz hg17 human genome (National Center for Biotechnology build 35, May 2004). aCGH analysis was also carried out as previously described [23]. Briefly, ratio of intensity values from tumor and normal tissues was transformed to log2-space. Log ratio data were then subjected to a circular binary segmentation (CBS) algorithm to reduce the effect of noise. Following this, a CGHcall algorithm was used to give each segment an aberration label: normal, deletion, or amplification. All our aCGH data can be accessed through the GEO ID GSE19180. Previously published aCGH data (GSE9654) was downloaded to perform the analysis [20].

### FISH detection and data analysis

FISH detection and analysis was performed in 54 FFPE OS samples as previously described [24]. FISH was performed using the *VEGFA* probe(Empire Genomics, Buffalo, NY) for detection and the CEP 6 probe(Abbott Molecular, Abbott Park, IL) as the reference. The *VEGFA* probe was hybridized to the short arm of chromosome 6(6p12), producing an orange signal, the intensity of which represented the *VEGFA* gene copy number. The CEP 6 probe was hybridized to the chromosome 6 centromere (6p11.1-q11.1), producing a green signal.

FISH results were interpreted independently and blinded by two pathologists [25]. Copy number alterations in which >90% of nuclei showed hybridization signals were considered informative. An informative case was considered *VEGFA* amplification if the ratio of orange to green signals was greater than 1 and there were more than two orange and green signals in each single tumor cell. If the ratio was equal to 1 and there were more than two green and orange signals in each single tumor cell, the case was considered to have increased *VEGFA* gene copy number. A case with a ratio lower than 1 or with only two green and orange signals in each single tumor cell was considered to have no *VEGFA* amplification [24].

### IHC analysis

Fifty-four representative FFPE sections were obtained for IHC staining as previously described [14,23,24]. Antibodies for WWOX, RUNX2 and VEGFA were purchased from Abcam (Abcam company, Cambridge, UK) with dilutions of 1:500, 1:100 and 1:100, respectively. Skin tissue, fetal cartilage from abortion tissue and breast carcinoma tissue served as positive controls for WWOX, RUNX2 and VEGFA staining, respectively. For negative controls, primary antibodies were substituted by PBS.

Two pathologists, blinded to the clinical information, evaluated and scored the IHC staining. Scoring of cytoplasmic WWOX and VEGFA staining, and of nuclear RUNX2 staining, was based on the staining intensity and extent. Microscopically, each section was observed randomly within 10 high-power fields(40×), each of which included 100 cells. First, staining extent was evaluated according to the proportion of positive tumor cells: 0% (score 0), <10% (score 1), 11‒25% (score 2), 26‒50% (score 3), 51‒75% (score 4) and > 75% (score 5). Then staining intensity was scored: no cell stain (score 0), yellow (score 1), tan (score 2) and brown (score 3). Final scores were calculated by multiplying intensity and extent scores and the results were divided as follows: negative ("-", score 0‒1), weak positive (" + ", score 2‒4), moderate positive ("++", score 5‒9) and strong positive ("+++",score 10‒15). For further study, staining results were also grouped into negative and positive (including weak positive, moderate positive and strong positive).

### Statistical analysis

We adopted SPSS version 16.0 for Windows to analyze the data. Student´s t-test or ANOVA was used to compare means, and frequencies were compared by means of the Chi-Square test. Survival analysis was carried out to inspect relevance between survival rate and expression using the Kaplan–Meier method and log-rank test. Correlations between the WWOX, RUNX2 and VEGFA genes and protein expression were assessed using Spearman’s test. Two-tailed *P* values less than 0.05 were considered to be statistically significant.

## Results

### Deletion of *WWOX* and frequent amplification of *RUNX2* and *VEGFA* genes in human OS

**Figure 1:**
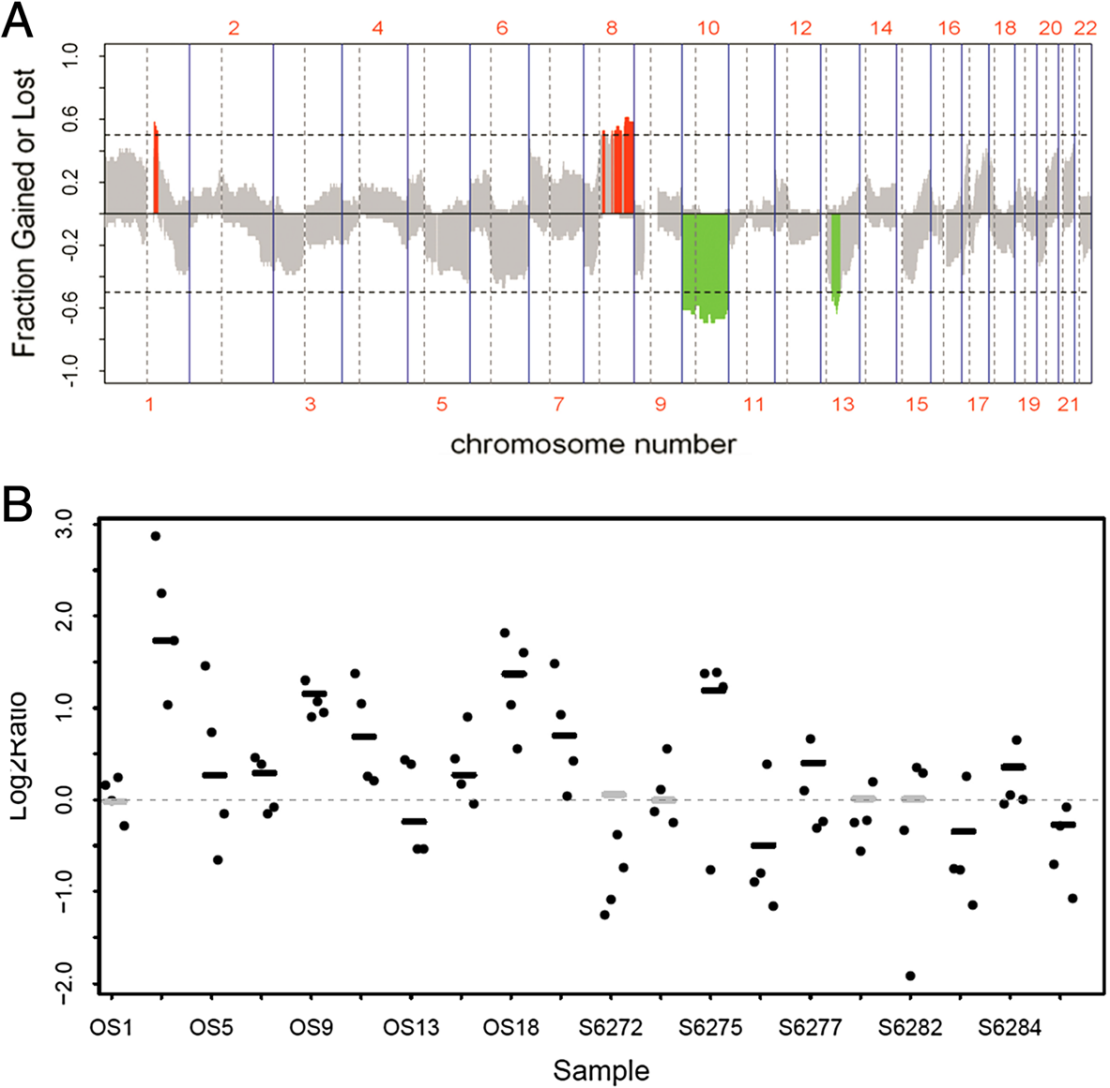
**Chromosomal and gene aberrations in 20 human osteosarcoma samples and the status of *RUNX2 *gene copy number aberrations (CNAs). A**. The recurrence pattern of CNAs in 20 human OS samples are illustrated in 2 microarray-based comparative genomic hybridization (aCGH) datasets (GSE19180and GSE9654). The x-axis indicates chromosome numbers and the y-axis indicates the aberration frequency of gains (positive)and losses (negative) for each measured aCGH probe, arranged based on their genomic coordinates along the x-axis. Dashed lines indicate the thresholds for significant recurrent aberrations. Measured sequences with aberration frequency that exceeded the thresholds are color-coded to emphasize the locations of significantly recurrent aberrations (red indicates significantly recurrent amplification; green, significantly recurrent deletion; grey, non-significant recurrence of aberrations). **B**. *RUNX2* gene copy number aberrations in OS. Sample IDs of the 20 OS samples in aCGH datasets GSE9654 and GSE19180 are indicated on the bottom. OS1–19 represent the case IDs in GSE9654 and S6272–6285 represent the case IDs in GSE19180. Scatters denote copy number change of the *RUNX2* gene. Lines in black and grey color denote the regional copy number value estimated by the circular binary segmentation (CBS) algorithm. Black lines denote significant amplification or deletion, whereas grey lines denote non-significant amplification or deletion. Twelve samples show amplification of the *RUNX2* gene.

Analysis of gene copy number alteration in 10 OS samples (GSE 19180) detected several chromosome genetic aberrations, including amplification of 1p35,1q23.1–1q21.1, 6p22.1–6p21.31, and 19p13.11–p13.2 and deletion of 5q12.3–5q13.2, 5q14.3–5q22.2, and 13q13.2–13p14.3 [23].These findings were validated by analyzing genetic aberrations in previously published aCGH data (GSE9654, 10 human OS samples) [20]. The overall recurrent gene copy alteration patterns of these two independent populations in two different countries (China and Canada) were very similar, suggesting that OS from diverse populations shares common genetic alterations at the gene copy level [23]. We therefore pooled the two aCGH data sets (total 20 OS) to perform further analysis (Figure 1A). Notably, the 12q13-15 amplification reported in low-grade OS was not significant in either dataset, nor were the *MDM2* and *CDK4* genes located in this region [26,27].

Analysis of the two aCGH data sets, GSE19180 and GSE 9654, identified a 30% (6/20) deletion rate of the *WWOX* gene in 20 human OS patients (3 cases from GSE19180 and 3 cases from GSE 9654) [23]. Furthermore, significant amplification of the *RUNX2* gene copy number was observed in 11 OS patients with an amplification frequency of 55% (11/20; 3 cases from GSE19180 and 8 cases from GSE 9654) (Figure 1B). For the *VEGFA* gene, we identified a 60% (12/20) amplification frequency (6 cases from GSE19180 and 6 cases from GSE 9654) in 20 OS patients [24].

**Figure 2:**
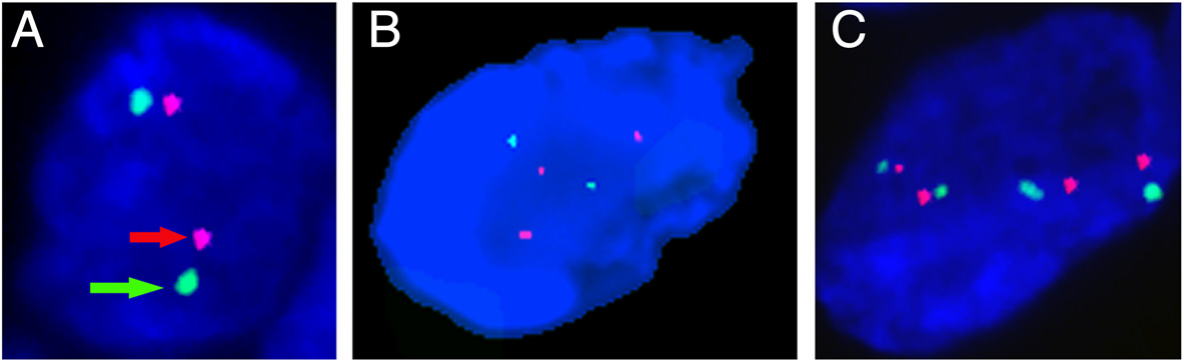
**Detection and validation of increased *VEGFA *gene copy number by FISH in OS.** Using FISH detection, orange represents *VEGFA* probe signal (red arrows) and therefore VEGFA copy number, whilst green represents CEP 6 reference probe signal located in the centromere of chromosome 6 (green arrows). When VEGFA/CEP 6 signal ratios were equal to or higher than 1, and when more than two gene copies of *VEGFA* were found per cell in more than 90% of OC cells, increased *VEGFA* gene copy number was recorded. Two patterns of increased *VEGFA* copy number exist in OS, focally or in larger fragment forms (polysomy). **A**: No copy number aberration of *VEGFA* gene, **B**: focally increase *VEGFA* gene copy number, **C**: increased copy number of *VEGFA* gene in larger fragment form (polysomy).

To validate the aCGH analysis, FISH was used to detect *VEGFA* gene status in 54 human FFPE OS tissues. We detected increased *VEGFA* gene copy number in 31 of 47 evaluable samples (65.9%) in either focal or large fragment forms (FISH results were not available for seven samples because there was insufficient tissue or loss of tissue during the pretreatment process) (Figure 2) [24]. From six samples showing increased *VEGFA* gene copy numbers based on the aCGH method (GSE 19180), FISH detected five samples showing increased *VEGFA* gene copy number. These two methods showed a good level of concordance (*P* = 0.048, *r* = 0.816).

To analyze possible interaction between *WWOX*, *RUNX2* and *VEGFA* gene copy number aberrations, we applied Spearman’s correlation test. However, there was no significant correlation between any two genes.

### Loss of WWOX protein expression, elevated RUNX2 and VEGFA protein expression in osteosarcoma tissues and their correlation

**Figure 3:**
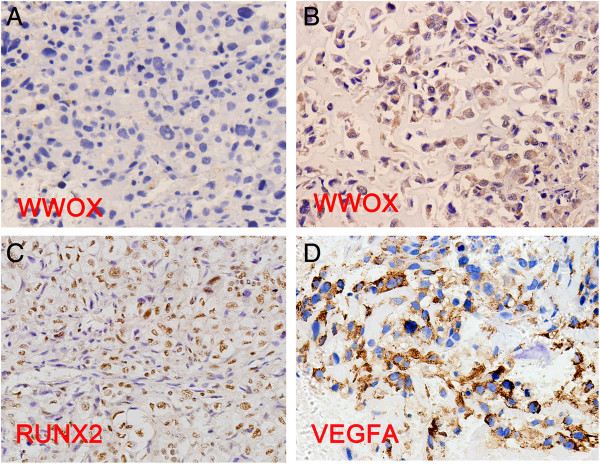
**Protein expression of WWOX, RUNX2 and VEGFA in human OS tissues by IHC (magnification, 40×). A**: negative expression of WWOX protein; **B**: strong positive expression of WWOX protein; **C**: strong positive expression of RUNX2 protein; **D**: strong positive expression of VEGFA protein.

WWOX and VEGFA protein expression was predominantly located in the cytoplasmic compartment with RUNX2 expression located in the cell nuclei (Figure 3). Frequent loss of WWOX protein expression (Figure 3A) was recorded in 61.1% (33/54) of samples with a positive WWOX expression rate in 38.9% (21/54) of samples (Figure 3B) [23]. RUNX2 protein expression was detected in 48.1% (26/54) of samples, including RUNX2 (+): 24.1% (13/54), (++): 5.6% (3/54) and (+++): 18.5% (10/54) (Figure 3C). VEGFA protein expression was detected in 75.9% (41/54) of samples, including VEGFA(+): 40.7% (22/54), (++): 11.1% (6/54) and (+++): 24.1% (13/54) (Figure3D).

Although no statistically significant association was found between WWOX expression and clinical pathologic factors, including sex, age, pTNM stage, recurrence, metastasis and survival, we found increased RUNX2 and VEGFA expression was significantly associated with age (*P* = 0.027) and sex(*P* = 0.012), respectively (Table 1).

Compared with increased *VEGFA* gene copy number, detected using FISH analysis, we identified a significant positive association between increased *VEGFA* gene copy number (65.9%, 31/47) and increased VEGFA protein expression (75.9%, 41/54) (*P* = 0.022) (Table 2). However, no significant correlation was detected between gene copy number (GSE 19180, 10 OS samples) and protein expression for WWOX (n = 10) and RUNX2 (n = 8) (IHC assay of RUNX2 protein expression was performed in 9 cases in which fresh samples were used in the aCGH analysis. One case which had aCGH data was excluded from IHC assay because of the sample was insufficient. For WWOX protein expression, only 8 cases could be used in the IHC assay. Therefore, we analyzed copy number alteration of the *WWOX* gene and its protein expression in 8 cases only) (Table 2). Negative RUNX2 protein expression was observed in all 3 OS samples with *RUNX2* amplification identified using aCGH detection (GSE 19180) (Table 2). Interestingly, the FFPE samples used for the IHC assay in these 3 cases were obtained from post-chemotherapy tissues, therefore this likely reflects the influence of chemotherapy on RUNX2 expression [14].

**Table 2:** Correlation between gene copy number aberration and protein expression of WWOX, RUNX2 and VEGFA

Protein expression*	***WWOX *** **deletion (detected by aCGH in 10 samples, GSE 19180)**		***P *** **value**	***RUNX2 *** **amplification (detected by aCGH in 10 samples, GSE 19180)**		***P *** **value**
	**No (%)**	**Yes (%)**		**No(%)**	**Yes(%)**	
Negative	2 (40.0)	3 (100)	0.237	3 (50.0)	3 (100)	0.325
Weak positive	1 (20.0)	0 (0)		0 (0)	0 (0)	
Moderate positive	0 (0)	0 (0)		2 (33.3)	0 (0)	
Strong positive	2 (40.0)	0 (0)		1 (16.7)	0 (0)	
VEGFA protein expression (detected by IHC in 54 cases)	Increased *VEGFA* gene copy number (detected by FISH in 54 cases)**			χ^2^	*P* value	
	No (%)	Yes (%)				
Negative	7 (43.8)	2 (6.5)		9.639	0.022***	
Weak positive	5 (31.2)	15 (48.4)				
Moderate positive	1 (6.2)	5 (16.1)				
Strong positive	3 (18.8)	9 (29.0)				

*IHC assay of RUNX2 protein expression was performed in nine cases from which fresh samples were used in the aCGH analysis. One aCGH data case was excluded from RUNX2 IHC analysis because of insufficient sample. For WWOX protein expression, only eight cases were used in the IHC assay.

**FISH results were unavailable for seven samples because the samples were insufficient and because of loss of tissues during the pretreatment process.

* **Significantly different *P* < 0.05.

In 39.4% (13/33) of OS samples with negative WWOX expression, RUNX2 was overexpressed, whereas 60.6% of samples with WWOX expression loss remained RUNX2 negative. However, statistical analysis failed to identify any significant inverse association between WWOX and RUNX2 protein expression (*P* = 0.073, *r* = 0.246) (Table 3). IHC analysis revealed 40.7% (22/54) concurrent positive expression between RUNX2 and VEGFA in OS samples (Table 3). Furthermore, Spearman’s correlation coefficient test showed a significant concordant relationship between RUNX2 and VEGFA (*P* = 0.008, *r* = 0.359) (Table 3).

**Table 3:** **Correlation between RUNX2 expression and WWOX/VEGFA expression in OS**, **detected by IHC in 54 cases**

**RUNX2**	**N**	**WWOX**				**VEGFA**			
		**-**	**+**	**++**	**+++**	**-**	**+**	**++**	**+++**
-	28	20	5	1	2	9	13	2	4
+	13	7	2	3	1	2	8	0	3
++	3	2	0	0	1	1	0	0	2
+++	10	4	3	2	1	1	1	4	4
Total	54								

Spearman’s correlation coefficient: r = 0.246, *P* = 0.073(RUNX2 and WWOX); r = 0.359, *P* = 0.008(RUNX2 and VEGFA).

## Discussion

OS is a malignant tumor of bone tissue with unknown etiology, and the survival rate has failed to improve since the introduction of chemotherapy [28]. In this study, we present genetic and molecular alterations and key associations between WWOX, RUNX2 and VEGFA expression in human OS. Our data show increased *RUNX2* and *VEGFA* gene copy number and protein elevation in human OS. Although significant reverse correlation was not observed between WWOX and RUNX2, we identified positive correlation of RUNX2 and VEGFA, suggesting both increased *VEGFA* gene copy number and RUNX2 overexpression facilitate increased expression of VEGFA, a key factor in tumor angiogenesis.

In the present study, we found no significant positive correlation between *WWOX* gene deletion (by aCGH) and reduced WWOX protein expression (by IHC) in OS. The result could stem from the low sample number in present study. Furthermore, there are several other factors involved the aberrant WWOX protein expression. According to previous reports, loss or attenuation of WWOX protein expression frequently results following abnormal mRNA splicing, missing exons, LOH and hypermethylation in the *WWOX* gene [4-9]. Furthermore, treatment such as chemotherapy might affect WWOX protein expression [14,29]. Aqeilan and colleagues showed that WWOX levels frequently increase in tumors resected following chemotherapy when compared with their primary biopsies. For these tumors, chemotherapy appears to induce tumor cell normalization rather than death, accompanied by restoration of WWOX expression [14,29]. The present study shows that OS samples with *WWOX* gene deletion loose protein expression, suggesting that *WWOX* gene copy number alteration also remains an important mechanism in the aberration of WWOX protein expression.

Consistent with previous reports, our data found *RUNX2* gene amplification and overexpression of its protein to be common in OS [21]. However, we found negative RUNX2 protein expression in three samples with positive *RUNX2* gene amplification. Closer assessment of these samples revealed they were obtained from post-chemotherapy tissues, suggesting chemotherapy may affect RUNX2 expression. Further evidence for this was found by Aqeilan and colleagues [14]. In their study, IHC analysis of 56 OS cases revealed 60% (12/20) pre-treatment biopsies were positive for RUNX2. However, only 16% (4/25) post-treatment resections were positive for RUNX2. Paired pre-treatment biopsy and post-treatment resections were available for 12 OS patients. Eight biopsies were RUNX2 positive and in all 8 cases (100%) these were RUNX2 negative post-treatment [14]. We therefore hypothesize that negative expression of RUNX2 protein in samples with *RUNX2* gene amplification might due to post-treatment. Pre-treatment tissues would be required to perform such analysis and support this hypothesis in future studies.

WWOX is known to suppress transactivation of RUNX2 by association with the first WW-domain, therefore expression of RUNX2 can be promoted in the absence of WWOX protein [13]. Kyle and colleagues report an inverse correlation between WWOX and RUNX2 expression in WWOX-deficient mice and OS cell lines [14]. In the present study, no significant correlation was observed between WWOX and RUNX2 expression. In fact, the inverse relationship between these factors observed by Kyle and colleagues was not evident when performing paired comparisons, similarly to our study results [14]. Thus, the relationship between WWOX and RUNX2 expression in OS remains unclear and warrants further investigation.

RUNX2 was previously reported as an essential component for the stimulation of VEGFA transcription during bone organogenesis [22]. In the present study, IHC analysis revealed a significant positive relationship between RUNX2 and VEGFA protein expression. These data suggestRUNX2 overexpression can induce increased VEGFA expression. VEGFA is known as a target of RUNX2 [22]. These two molecules are synergistic in the process of angiogenesis, which is in accordance with a previous report [22]. At the same time, the significant relation between increased *VEGFA* gene copy number and increased VEGFA expression suggests that increased *VEGFA* gene copy number is also important in VEGFA protein expression. These data provide powerful evidence that increased *VEGFA* gene copy number and RUNX2 overexpression facilitate increased expression of VEGFA, a key factor in tumor angiogenesis.

## Conclusions

The present study found no significant correlation between WWOX, RUNX2, and VEGFA genes with respect to gene copy number aberration. Significant association was observed between increased *VEGFA* gene copy number and protein expression, whilst *WWOX* and *RUNX2* genes failed to show such association. This may be due to the regulation of other factors in addition to the effect of pre-treatment. No significant association was observed between WWOX and RUNX2 protein expression, but a significantly positive relationship was observed between RUNX2 and VEGFA protein expression. WWOX, RUNX2 and VEGFA functional crosstalk may be essential for the pathogenesis and angiogenesis of OS, and this pathway might provide a new molecular basis for targeted RUNX2-VEGFA therapy in OS patients.

## Competing interests

The authors have declared no conflicts of interest.

## Authors’ contributions

JY, LZ and WT carried out the molecular genetic studies, participated in the aCGH, IHC and FISH assays and drafted the manuscript. HZ, ZL and GW participated in the design of the study and performed statistical analysis. ZL revised the manuscript. JY and KC conceived the study, participated in its design and coordination and helped draft the manuscript. All authors have read and approved the final manuscript.

## Pre-publication history

The pre-publication history for this paper can be accessed here:

http://www.biomedcentral.com/1755-8794/6/56/prepub

## References

[B1] Ottaviani G Jaffe N The etiology of osteosarcoma Canc Treat Res 2009 6 15 32 10.1007/978-1-4419-0284-9_2 20213384

[B2] Lim G Karaskova J Vukovic B Bayani J Beheshti B Bernardini M Squire JA Zielenska M Combined spectral karyotyping, multicolor banding, and microarray comparative genomic hybridization analysis provides a detailed characterization of complex structural chromosomal rearrangements associated with gene amplification in the osteosarcoma cell line MG-63 Canc Genet Cytogenet 2004 6 2 158 164 10.1016/j.cancergencyto.2004.01.016 15350306

[B3] Yang J Zhang W WWOX tumor suppressor gene Histol Histopathol 2008 6 7 877 882 18437686 10.14670/HH-23.877

[B4] Yendamuri S Kuroki T Trapasso F Henry AC Dumon KR Huebner K Williams NN Kaiser LR Croce CM WW domain containing oxidoreductase gene expression is altered in non-small cell lung cancer Canc Res 2003 6 4 878 881 12591741

[B5] Aqeilan RI Kuroki T Pekarsky Y Albagha O Trapasso F Baffa R Huebner K Edmonds P Croce CM Loss of WWOX expression in gastric carcinoma Clin Canc Res: Offic J Am Assoc Canc Res 2004 6 9 3053 3058 10.1158/1078-0432.CCR-03-0594 15131042

[B6] Park SW Ludes-Meyers J Zimonjic DB Durkin ME Popescu NC Aldaz CM Frequent downregulation and loss of WWOX gene expression in human hepatocellular carcinoma Br J Canc 2004 6 4 753 759 10.1038/sj.bjc.6602023 PMC2364795 15266310

[B7] Nunez MI Rosen DG Ludes-Meyers JH Abba MC Kil H Page R Klein-Szanto AJ Godwin AK Liu J Mills GB WWOX protein expression varies among ovarian carcinoma histotypes and correlates with less favorable outcome BMC Canc 2005 6 64 10.1186/1471-2407-5-64 PMC1173095 15982416

[B8] Iliopoulos D Guler G Han SY Johnston D Druck T McCorkell KA Palazzo J McCue PA Baffa R Huebner K Fragile genes as biomarkers: epigenetic control of WWOX and FHIT in lung, breast and bladder cancer Oncogene 2005 6 9 1625 1633 10.1038/sj.onc.1208398 15674328

[B9] Qin HR Iliopoulos D Semba S Fabbri M Druck T Volinia S Croce CM Morrison CD Klein RD Huebner K A role for the WWOX gene in prostate cancer Canc Res 2006 6 13 6477 6481 10.1158/0008-5472.CAN-06-0956 16818616

[B10] Chang NS Pratt N Heath J Schultz L Sleve D Carey GB Zevotek N Hyaluronidase induction of a WW domain-containing oxidoreductase that enhances tumor necrosis factor cytotoxicity J Biol Chem 2001 6 5 3361 3370 10.1074/jbc.M007140200 11058590

[B11] Aqeilan RI Donati V Palamarchuk A Trapasso F Kaou M Pekarsky Y Sudol M Croce CM WW domain-containing proteins, WWOX and YAP, compete for interaction with ErbB-4 and modulate its transcriptional function Canc Res 2005 6 15 6764 6772 10.1158/0008-5472.CAN-05-1150 16061658

[B12] Gaudio E Palamarchuk A Palumbo T Trapasso F Pekarsky Y Croce CM Aqeilan RI Physical association with WWOX suppresses c-Jun transcriptional activity Canc Res 2006 6 24 11585 11589 10.1158/0008-5472.CAN-06-3376 17178850

[B13] Aqeilan RI Hassan MQ de Bruin A Hagan JP Volinia S Palumbo T Hussain S Lee SH Gaur T Stein GS The WWOX tumor suppressor is essential for postnatal survival and normal bone metabolism J Biol Chem 2008 6 31 21629 21639 10.1074/jbc.M800855200 18487609 PMC2490770

[B14] Kurek KC Del Mare S Salah Z Abdeen S Sadiq H Lee SH Gaudio E Zanesi N Jones KB DeYoung B Frequent attenuation of the WWOX tumor suppressor in osteosarcoma is associated with increased tumorigenicity and aberrant RUNX2 expression Canc Res 2010 6 13 5577 5586 10.1158/0008-5472.CAN-09-4602 PMC3037996 20530675

[B15] Pratap J Lian JB Javed A Barnes GL van Wijnen AJ Stein JL Stein GS Regulatory roles of Runx2 in metastatic tumor and cancer cell interactions with bone Canc Metastasis Rev 2006 6 4 589 600 10.1007/s10555-006-9032-0 17165130

[B16] Won KY Park HR Park YK Prognostic implication of immunohistochemical Runx2 expression in osteosarcoma Tumori 2009 6 3 311 316 19688969 10.1177/030089160909500307

[B17] Lu XY Lu Y Zhao YJ Jaeweon K Kang J Xiao-Nan L Ge G Meyer R Perlaky L Hicks J Cell cycle regulator gene CDC5L, a potential target for 6p12-p21 amplicon in osteosarcoma Mol Canc Res: MCR 2008 6 6 937 946 10.1158/1541-7786.MCR-07-2115 PMC2693718 18567798

[B18] Man TK Lu XY Jaeweon K Perlaky L Harris CP Shah S Ladanyi M Gorlick R Lau CC Rao PH Genome-wide array comparative genomic hybridization analysis reveals distinct amplifications in osteosarcoma BMC Canc 2004 6 45 10.1186/1471-2407-4-45 PMC514550 15298715

[B19] Lau CC Harris CP Lu XY Perlaky L Gogineni S Chintagumpala M Hicks J Johnson ME Davino NA Huvos AG Frequent amplification and rearrangement of chromosomal bands 6p12-p21 and 17p11.2 in osteosarcoma Gene Chromosome Canc 2004 6 1 11 21 10.1002/gcc.10291 14603437

[B20] Squire JA Pei J Marrano P Beheshti B Bayani J Lim G Moldovan L Zielenska M High-resolution mapping of amplifications and deletions in pediatric osteosarcoma by use of CGH analysis of cDNA microarrays Gene Chromosome Canc 2003 6 3 215 225 10.1002/gcc.10273 14506695

[B21] Sadikovic B Yoshimoto M Chilton-MacNeill S Thorner P Squire JA Zielenska M Identification of interactive networks of gene expression associated with osteosarcoma oncogenesis by integrated molecular profiling Hum Mol Genet 2009 6 11 1962 1975 10.1093/hmg/ddp117 19286668

[B22] Zelzer E Glotzer DJ Hartmann C Thomas D Fukai N Soker S Olsen BR Tissue specific regulation of VEGF expression during bone development requires Cbfa1/Runx2 Mech Dev 2001 6 1–2 97 106 11472838 10.1016/s0925-4773(01)00428-2

[B23] Yang J Cogdell D Yang D Hu L Li H Zheng H Du X Pang Y Trent J Chen K Deletion of the WWOX gene and frequent loss of its protein expression in human osteosarcoma Canc Lett 2010 6 1 31 38 10.1016/j.canlet.2009.09.018 19896763

[B24] Yang J Yang D Sun Y Sun B Wang G Trent JC Araujo DM Chen K Zhang W Genetic amplification of the vascular endothelial growth factor (VEGF) pathway genes, including VEGFA, in human osteosarcoma Cancer 2011 6 21 4925 4938 10.1002/cncr.26116 21495021 PMC3465081

[B25] Schildhaus HU Heukamp LC Merkelbach-Bruse S Riesner K Schmitz K Binot E Paggen E Albus K Schulte W Ko YD Definition of a fluorescence in-situ hybridization score identifies high- and low-level FGFR1 amplification types in squamous cell lung cancer Modern Pathol: Offic J Unit States Can Acad Pathol Inc 2012 6 11 1473 1480 10.1038/modpathol.2012.102 PMC4089812 22684217

[B26] Dujardin F Binh MB Bouvier C Gomez-Brouchet A Larousserie F Muret A Louis-Brennetot C Aurias A Coindre JM Guillou L MDM2 and CDK4 immunohistochemistry is a valuable tool in the differential diagnosis of low-grade osteosarcomas and other primary fibro-osseous lesions of the bone Modern Pathol: Offic J Unit States Can Acad Pathol Inc 2011 6 5 624 637 10.1038/modpathol.2010.229 21336260

[B27] Lopes MA Nikitakis NG Ord RA Sauk J Jr Amplification and protein expression of chromosome 12q13-15 genes in osteosarcomas of the jaws Oral Oncol 2001 6 7 566 571 10.1016/S1368-8375(00)00130-5 11564577

[B28] Longhi A Errani C De Paolis M Mercuri M Bacci G Primary bone osteosarcoma in the pediatric age: state of the art Canc Treat Rev 2006 6 6 423 436 10.1016/j.ctrv.2006.05.005 16860938

[B29] Del Mare S Kurek KC Stein GS Lian JB Aqeilan RI Role of the WWOX tumor suppressor gene in bone homeostasis and the pathogenesis of osteosarcoma Am J Canc Res 2011 6 5 585 594 PMC3124638 21731849

